# Biosecurity interceptions of an invasive lizard: origin of stowaways and human-assisted spread within New Zealand

**DOI:** 10.1111/eva.12002

**Published:** 2012-09-03

**Authors:** David G Chapple, Anthony H Whitaker, Stephanie N J Chapple, Kimberly A Miller, Michael B Thompson

**Affiliations:** 1 School of Biological Sciences, Monash UniversityClayton, Vic., Australia; 2 Division of Sciences, Museum VictoriaMelbourne, Vic., Australia; 3 Allan Wilson Centre for Molecular Ecology and Evolution, School of Biological Sciences, Victoria University of WellingtonWellington, New Zealand; 4 Orinoco, Motueka, New Zealand; 5 Department of Zoology, University of MelbourneMelbourne, Vic., Australia; 6 School of Biological Sciences, University of SydneySydney, NSW, Australia

**Keywords:** Australia, biological invasion, biosecurity, human-assisted dispersal, mitochondrial DNA, New Zealand, trade routes, unintentional introduction

## Abstract

Globalization, and the resultant movement of animals beyond their native range, creates challenges for biosecurity agencies. Limited records of unintentional introductions inhibit our understanding of the trade pathways, transport vectors and mechanisms through which hitchhiker organisms are spread as stowaways. Here, we adopt a phylogeographic approach to determine the source and human-mediated dispersal pathways of New Zealand's only invasive lizard, the delicate skink (*Lampropholis delicata*), intercepted by biosecurity agencies in New Zealand. Biosecurity agencies correctly predicted the source region of 77% of stowaways, which were usually solitary adults, arriving via air or sea pathways during the cooler months, evading initial border checks and alive when detected. New arrivals from Australia comprised 16% of detections originating from the region between Brisbane and Sydney. Our analyses indicate human-mediated dispersal has driven the post-border spread of *L. delicata* within New Zealand. Propagule pressure was substantially greater for *L. delicata* compared with the noninvasive, congeneric *Lampropholis guichenoti*. Our results highlight the transport pathways, spread mechanisms, and stowaway characteristics of *Lampropholis* lizards entering New Zealand, which could enhance current biosecurity protocols and prevent the establishment of additional lizard species.

## Introduction

For centuries, human activities have resulted in the unintentional movement of animals to regions outside of their native range (Elton 1958; Lockwood et al. 2007; Richardson 2011). The vast majority of stowaways fail to establish in the new environment, but a select subset manage to thrive, spread throughout the recipient region and become invasive (Blackburn et al. 2011; Chapple et al. 2012a,b). Invasive species are a leading threat to biodiversity owing to their potentially adverse impacts on native species and ecosystems (Gurevitch and Padilla 2004; Didham et al. 2005) and may impose substantial economic costs as a result of their impacts on agriculture, forestry, fisheries, tourism and human health (Pimentel et al. 2001, 2005). Once established, introduced species are extremely difficult and sometimes impossible to control or eradicate (Mack et al. 2000; Lockwood et al. 2007; Kraus 2009). Thus, prevention is better than cure, and biosecurity measures that reduce the likelihood of the transportation and initial establishment of species are the most effective strategy for management of invasive species (Mack et al. 2000; Meyerson and Reaser 2002; Ruiz and Carlton 2003; Toy and Newfield 2010).

The rapid proliferation in international trade associated with our increasing globalization represents a significant challenge for biosecurity agencies (Meyerson and Mooney 2007; Hulme 2009). Propagule pressure, the number of individuals of a particular species arriving in a recipient region, is an important determinant of both establishment and invasion success (Lockwood et al. 2005; Simberloff 2009) and will increase as the number or frequency of trade pathways rises in species proficient at human-assisted dispersal (Chapple et al. 2012a). Biosecurity measures aim to prevent animals entering transport vectors, intercept stowaways at the border or manage post-border incursions (Meyerson and Reaser 2002; Ruiz and Carlton 2003). Effective biosecurity protocols require knowledge of the transport hubs and initial entry ports through which the stowaways pass, an understanding of the transport vectors and dispersal mechanisms used and accurate predictions (e.g. based on species’ traits and the climatic suitability of the recipient region) of which stowaways represent potential invaders (Hayes and Barry 2008; Hulme et al. 2008; Hulme 2009; Kraus 2009). This information is exceedingly difficult to obtain for most unintentional introductions, as documentation associated with the movement of stowaways is usually limited (Allen et al. 2006; Kraus 2009) and introduction pathways (and sometimes accurate identification of the species itself) must be inferred using trade records and/or genetic analyses (Kolbe et al. 2004; Muirhead et al. 2008; Estoup and Guillemaud 2010).

Despite its isolation, New Zealand is one of the more heavily invaded countries in the world, and the impact of exotic species on the native biota has been acknowledged for decades (Elton 1958; Allen et al. 2006; Lee et al. 2006). This has contributed to New Zealand developing the world's most comprehensive approach to biosecurity (Meyerson and Reaser 2002; Hayden and Whyte 2003), which is supported by unified legislation (*Biosecurity Act 1993*) and a well-resourced government agency [Ministry for Primary Industries (MPI); known prior to April 2012 as the Ministry of Agriculture and Forestry, Biosecurity New Zealand]. Identifying arrival pathways is simplified in New Zealand as there are no land borders and stowaway animals can arrive only via air and sea transport routes. Importantly, MPI maintains a detailed database of exotic stowaways that are detected at the border and immediately post-border and also documents the post-border movements of taxa specified as Unwanted Organisms under the *Biosecurity Act 1993*. This is an invaluable resource that has been used in risk analysis and the investigation of introduction pathways of invertebrate stowaways, particularly ants (Armstrong and Ball 2005; Lester 2005; Ward et al. 2005, 2006; Ball and Armstrong 2006; Corin et al. 2007, 2008).

With the tightening of biosecurity protocols in New Zealand, the focus has switched from taxa that are principally introduced through deliberate means (e.g. birds, mammals, freshwater fish) to those that are generally introduced unintentionally (e.g. invertebrates, squamate reptiles, frogs) (Allen et al. 2006; Kraus 2009). Even rigorous biosecurity screening may fail to detect a substantial proportion of stowaways hidden in freight and cargo (Allen et al. 2006; Ward et al. 2006; Toy and Newfield 2010), which is a significant concern given the exponential increase in the inadvertent transportation of squamate reptiles over the last century (Kraus 2009). During the last century, 92 lizard species (from across 11 families) have been intercepted entering New Zealand, many of which are invasive elsewhere, including some of the world's most invasive reptiles (e.g. Asian house gecko, *Hemidactylus frenatus*; mourning gecko, *Lepidodactylus lugubris*) (Gill et al. 2001; Lever 2003; Kraus 2009; A. H. Whitaker unpublished data). However, only one lizard species, the delicate skink (*Lampropholis delicata*; also known as the rainbow skink), has successfully established and become invasive in New Zealand (Gill et al. 2001; Lever 2003; Kraus 2009).

**Figure 1:**
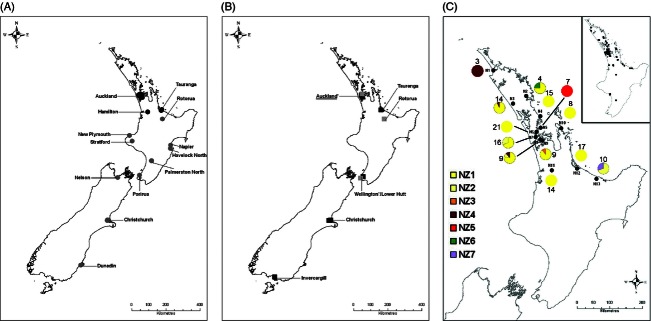
(A, B) Location of *Lampropholis* specimens intercepted by Ministry for Primary Industries. The four types of detection are indicated: local *Lampropholis delicata* within the established range (black circles), human-assisted dispersal of *L. delicata* within New Zealand to an area outside the established range (grey circles), new arrivals of *L. delicata* direct from Australia (black squares), and the location of *Lampropholis guichenoti* interceptions (grey squares). Underlined city names indicate that both species have been intercepted at this location. (C) Distribution and relative abundance of mitochondrial haplotypes across the established range of *L. delicata* in New Zealand (adapted from Chapple et al. 2012c). The population numbers refer to those provided in Table S3. The sample size for each population is indicated. The current distribution of *L. delicata* in New Zealand is provided in the inset figure (adapted from the New Zealand Department of Conservation Herpetofauna database records). Note that the Palmerston North population did not become established until approximately 2007.

*Lampropholis delicata* is a small-sized skink [adult snout-vent length (SVL) 35–51 mm] that is native to eastern Australia (Wilson and Swan 2010). It was first detected in New Zealand in the mid-1960s at the Otahuhu railyards in south Auckland and is thought to have arrived as a stowaway in a shipment of wooden railway sleepers (Lever 2003; Fig. 1). We have provided support for this hypothesis previously, showing that the established New Zealand populations of *L. delicata* resulted from one or more successful introductions from a forestry region in inland northern New South Wales, near Tenterfield (Chapple et al. 2012c). Its introduction seems to have been localized to the Auckland region for approximately 15 years before rapidly spreading across the northern North Island (Lever 2003; Peace 2004; Chapple et al. 2012c; Fig. 1). It is still expanding its range in the North Island, with bioclimatic modelling indicating that it has the potential to spread throughout much of the North Island and into some regions of the South Island (Lever 2003; Peace 2004). *Lampropholis delicata* is also invasive in the Hawaiian Islands and Lord Howe Island (Lever 2003; Chapple et al. 2012c). It is thought to have rapidly displaced the resident moth skink (*Lipinia noctua*) soon after its arrival in Hawaii (Baker 1979; but see Fisher and Ineich 2012) and has the potential to adversely impact the diverse native New Zealand lizard fauna (approximately 100 species, Hitchmough et al. 2010) owing to its rapid maturity (approximately 1 year), high annual reproductive output, extreme population densities and ability to thrive in areas with human disturbance and introduced mammals (Lever 2003; Peace 2004).

Here we adopt a detailed phylogeographic approach to identify the introduction pathways and post-border spread of *L. delicata* in New Zealand. Specifically, we use mitochondrial sequence data from *L. delicata* intercepted by MPI to identify the Australian region of origin of each stowaway, the transport vector and port of entry for each detection, the characteristics of each stowaway and whether detections represent new arrivals direct from Australia or post-border movement of lizards within New Zealand. Ours is the first comprehensive analysis of the effectiveness of biosecurity protocols (Allen et al. 2006; Kraus 2009; Toy and Newfield 2010) and is only possible here owing to the availability of the MPI database of lizard interceptions, access to the voucher specimens from each detection and knowledge of the Australian source for the established *L. delicata* populations in New Zealand. Thus, *L. delicata* provides an ideal organism with which to examine the effectiveness of the current biosecurity protocols and assess the potential for further lizard species to become established in New Zealand in the future. As a first step towards this objective, we also examine the MPI interceptions of the congeneric *Lampropholis guichenoti* (garden skink), which is found in sympatry with *L. delicata* across most of their range in eastern Australia. Despite near identical biology and ‘opportunity’ for transportation (Chapple et al. 2011a, 2012a), *L. guichenoti* has never successfully established outside of Australia.

## Materials and methods

### Sample collection

**Figure 2:**
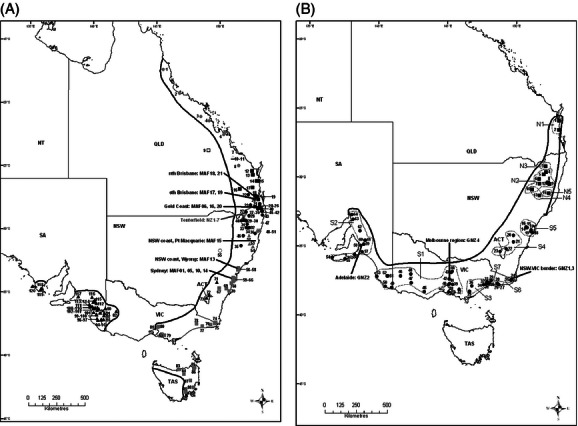
(A) Location of the *Lampropholis delicata* samples from the native range in eastern Australia. The source region(s) for the established New Zealand populations (grey text) and biosecurity interceptions (black text) are indicated (Table 1, Fig 3A). The population numbers refer to those presented in Table S2. The distribution of the nine major clades (Fig. 3A) is indicated: clade 1 (grey solid circles), clade 2 (hollow triangles), clade 3 (black solid squares), clade 4 (black solid circles), clade 5 (grey solid triangles), clade 6 (hollow squares), clade 7 (black solid triangles), clade 8 (hollow circles), clade 9 (grey solid squares). (B) Location of the *Lampropholis guichenoti* samples from the native range in eastern Australia. The source region for the biosecurity interceptions is indicated (Table 3, Fig. 3B). The population numbers refer to those presented in Table S4. The distribution of the northern (black squares) and southern lineages (black circles) is indicated, along with the clade distributions (Fig. 3B). For both maps: the approximate native distribution of each species is indicated by the solid line (adapted from Wilson and Swan 2010). NSW, New South Wales; NT, Northern Territory; QLD, Queensland; SA, South Australia; TAS, Tasmania, VIC, Victoria.

We obtained tissue samples from the *L. delicata* (*n* = 79) and *L. guichenoti* (*n* = 4) specimens intercepted by, or reported to, MPI between 2001 and 2008 (Fig. 1, Table S1). To distinguish between (i) animals of local origin from the established New Zealand population, (ii) human-assisted extra-limital spread within New Zealand and (iii) new arrivals into the country direct from Australia (and determine their origin), we used sequence data from Chapple et al. (2011b), which includes the entire native range of the species (238 samples from 120 populations; GenBank accession no.: HQ454791, JF438009-JF438483; Fig. 2A, Table S2) and included the seven haplotypes known to occur in the established *L. delicata* populations in New Zealand (Chapple et al. 2012c; GenBank accession no.: JF915805-JF915811; Table S3). We included *L. guichenoti* (Australian Museum NR2639; GenBank accession no.: EF567304, EU567769) and an Australian *Eugongylus*-lineage skink *Niveoscincus pretiosus* (Australian Museum NR391; GenBank accession no.: EF567726, EF567768) as outgroups.

For *L. guichenoti*, we used sequence data from Chapple et al. (2011c) from across its range in eastern Australia (123 samples from 64 populations; GenBank accession no.: HQ454789-HQ454913; Fig. 2B, Table S4) to identify the source region(s) for the individuals intercepted entering New Zealand. We used *L. delicata* (LDA124; Table S2) and *N. pretiosus* as outgroups in the *L. guichenoti* phylogenetic analyses.

### DNA extraction, amplification and sequencing

Total genomic DNA was extracted from liver, muscle or tail-tip samples using a Qiagen DNeasy Blood and Tissue Extraction Kit (Qiagen, Hilden, Germany). For *L. delicata*, we amplified and sequenced portions of two mitochondrial genes, *ND2* (approximately 600 bp) and *ND4* (approximately 700 bp), as outlined in Chapple et al. (2011b). For *L. guichenoti,* we sequenced the *ND4* mitochondrial gene as outlined in Chapple et al. (2011c). PCR products were purified using ExoSAP-IT (USB Corporation, Cleveland, OH, USA). The purified product was sequenced directly using a BigDye Terminator v3.1 Cycle Sequencing Kit (Applied Biosystems, Foster City, CA, USA) and then analysed on an ABI 3730XL capillary sequencer.

Sequence data were edited using Geneious v5.4 (Drummond et al. 2011) and aligned using the default parameters of Clustal W (Thompson et al. 1994) executed in mega 4 (Tamura et al. 2007). We translated all sequences to confirm that none contained premature stop codons. The haplotypes present in the *L. delicata* and *L. guichenoti* specimens intercepted by MPI were identified using DnaSP v5.10 (Librado and Rozas 2009) and were submitted to GenBank under the accession numbers JQ413190-JQ413223 (Table S1). Tamura-Nei (TrN) corrected genetic distances among haplotypes were calculated in mega.

### Phylogenetic analyses

We generated phylogenetic trees for both species using neighbour-joining (NJ), maximum-likelihood (ML) and Bayesian methods. For *L. delicata*, we used the haplotypes from the native range, established New Zealand populations and biosecurity interceptions. For *L. guichenoti*, we used the haplotypes from the native range and biosecurity interceptions to generate the phylogenetic trees. We used Modeltest 3.7 (Posada and Crandall 1998) for the ML and Bayesian analyses to identify the most appropriate model of sequence evolution based on the Akaike Information Criterion (AIC) criterion. Modeltest was also used to estimate base frequencies, substitution rates, the proportion of invariable sites (*I*) and the among-site substitution rate variation (*G*). These values were then used as settings in PhyML 3.0 (Guindon and Gascuel 2003) to generate ML trees with 500 bootstraps. NJ trees were generated in mega using the TrN model correction.

MrBayes 3.1.2 (Ronquist and Huelsenbeck 2003) was used to complete Bayesian analyses. For each species, we ran the full analysis twice, using four Markov chains per run. We ran the chains for five million generations to ensure sufficient sampling of tree space. The chain was sampled every 100 generations to obtain 50 000 sampled trees. The program Tracer 1.5 (Rambaut and Drummond 2007) was used to check for chain convergence. The first 25% of sampled trees were discarded as the burn-in phase and the last 37 500 trees were used to estimate the Bayesian posterior probabilities (PP). Bootstrap values (500 ML bootstraps) and Bayesian PP were used to assess branch support.

For *L. delicata*, animals that had haplotypes found in the established New Zealand populations (or from the same source region in inland northern NSW) were considered to be part of the local, invasive population in the country. Of these, those individuals detected outside of the established range in New Zealand were considered to represent instances of human-assisted movement of *L. delicata* within the country. The Australian origin for individuals determined to be new arrivals into New Zealand was established by identifying the most closely related haplotype(s) from the native range. A similar approach was used to determine the source region for *L. guichenoti* specimens intercepted entering New Zealand.

### Analysis of biosecurity records

Ministry for Primary Industries maintains a database of interceptions of exotic reptiles and amphibians. We analysed the records for *L. delicata* and *L. guichenoti* intercepted between 2001 and 2008. For each record, we compared the predicted origin (i.e. local New Zealand origin versus movement within the country versus new arrival from Australia) with that determined by our molecular data. For new introductions into New Zealand, we compared the origin within Australia predicted by import pathway data with that indicated by our genetic analyses. We analysed these new arrivals in terms of interception month, transport method (air, sea), cargo type (personal effects, shipping container), point of entry/interception, whether the detection occurred at the border or post-border, lizard body size (SVL; both species reach sexual maturity at approximately 35 mm; Wilson and Swan 2010) and whether the lizard was alive when detected. We completed a similar analysis for the *L. delicata* specimens transported from a location within the current New Zealand range to an area where it has yet to establish.

## Results

### *Lampropholis delicata* phylogenetic analyses

The edited alignment comprised 1221 characters (550 bp *ND2*, 671 bp *ND4*), of which 541 (44.3%) were variable and 413 (33.8%) were parsimony informative. For the ingroup only, the alignment contained 455 (37.3%) variable characters, of which, 393 (32.2%) were parsimony informative. Base frequencies were unequal (A = 0.326, T = 0.244, C = 0.310, G = 0.120), but a chi-square test confirmed the homogeneity of base frequencies among sequences (χ^2^ = 57.13, df = 582, *P* = 1.0).

**Figure 3:**
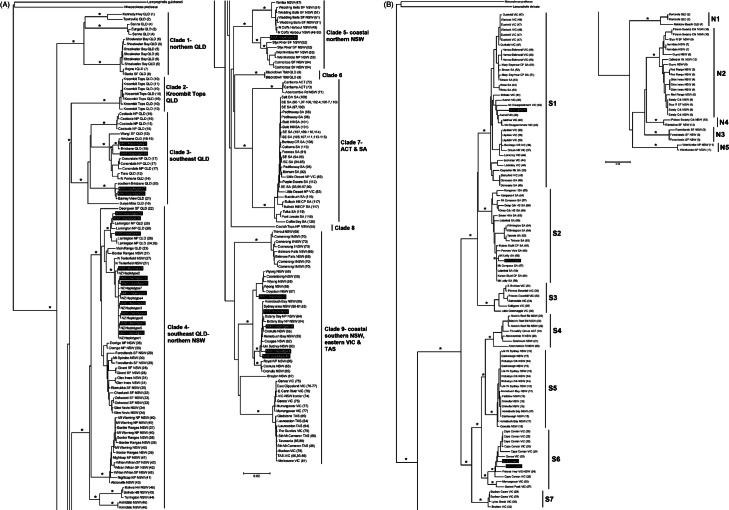
(A) Phylogenetic tree for *Lampropholis delicata*, based on 1221 bp of mitochondrial DNA (550 bp *ND2*, 671 bp *ND4*). The position of the haplotypes (highlighted in black) from the biosecurity interceptions are indicated, along with the haplotypes from the established populations in New Zealand (highlighted in grey). The population numbers listed in Table S2 are provided in parentheses. Nine major genetic clades are identified in *L. delicata* (as per Chapple et al. 2011b). (B) Phylogenetic tree for *Lampropholis guichenoti*, based on 671 bp of *ND4*. The position of the haplotypes (highlighted in black) from the biosecurity interceptions are indicated. The population numbers listed in Table S4 are provided in parentheses. The two lineages and main subclades identified in *L. guichenoti* are indicated [as per Chapple et al. 2011c). For both trees: The tree is split with the top half on the left. Measures of branch support (ML bootstrap values, Bayesian posterior probabilities (PP)] are shown only for the nodes for the main clades or lineages. The asterisks indicate the well-supported nodes (i.e. bootstraps >70, PP > 0.95).

The AIC from Modeltest supported the TrN+I+G substitution model as the most appropriate for our data set. Parameters estimated under this model were as follows: relative substitution rates (A↔C = 1.0000, A↔G = 30.3353, A↔T = 1.0000, C↔G = 1.0000, C↔T = 13.6134, relative to G↔T = 1.0000), proportion of invariable sites (0.4255) and gamma distribution shape parameter (0.8759). The topologies of the NJ, ML and Bayesian trees were almost identical; therefore, we present a phylogenetic tree with ML bootstrap (BS) values and Bayesian PP indicating branch support (Fig. 3A). The same nine well-supported, geographically nonoverlapping clades identified in Chapple et al. (2011b) were recovered (Fig. 3A).

### *Lampropholis delicata* interceptions: local residents, post-border dispersal within New Zealand, or new arrivals?

Determination of the haplotype evident in each intercepted *L. delicata* enabled us to categorize each individual as either part of the established New Zealand population, an instance of extra-limital dispersal within New Zealand or a new arrival from Australia. Twenty-one haplotypes were identified from the 79 *L. delicata* included in the MPI database (Fig. 3A, Table S1). The majority of the lizards (63 individuals) contained haplotypes that were identical to known haplotypes from the established range of the species in New Zealand [MAF02 = NZ4 (*n* = 4), MAF03 = NZ1 (*n* = 46), MAF04 = NZ6 (*n* = 2), MAF07 = NZ5 (*n* = 1), MAF08 = NZ3 (*n* = 6) and MAF09 = NZ2 (*n* = 4); Fig. 3A, Tables S1 and S3]. A further three lizards had haplotypes closely related (genetic distance 0.2–0.3%) to those known from the established range (MAF 11, *n* = 2; MAF12, *n* = 1) and likely represent previously undetected haplotypes from the same Tenterfield source region (Figs 2A and 3A, Table S1). Thus, 66 of the *L. delicata* detections were found to be animals of local New Zealand origin or the human-assisted movement of individuals from the established populations to regions beyond the species current range in the country (this includes all 58 predictions of a New Zealand origin in the MPI database; Table S1).

Our analyses indicated that at least 23 of the *L. delicata* records represented human-assisted movement from within its established New Zealand range to a location beyond its current distribution in the country (one additional record from Palmerston North appears to represent an emerging population; Fig. 1, Table 2). Twenty of these movements were correctly predicted from pathway data (Tables 2 and S1). These included extra-limital detections in locations in both the North Island [Havelock North (*n* = 1), New Plymouth (*n* = 2), Stratford (*n* = 1), Rotorua (*n* = 1), Napier (*n* = 2), Palmerston North (*n* = 6 or 7, depending the establishment date in the location), Porirua (*n* = 1)] and South Island [Nelson (*n* = 1), Christchurch (*n* = 6), Dunedin (*n* = 2)] (Fig. 1, Table 2). For the 21 detections where the freight movement information is available, 20 are known to have originated in the Auckland region and one from Waihi Beach in the western Bay of Plenty (Table 2).

Based on import pathway data, 16 of the 79 detections of *L. delicata* listed in the MPI data set were predicted to represent new arrivals into New Zealand direct from Australia (Table S1; for a further five detections, it was not possible to completely exclude an Australian origin). Our molecular data confirmed an Australian origin for 13 interceptions, all of them included within the 16 predicted in the MPI database to represent new introductions (a 81.3% success rate; the five equivocal detections were confirmed as local New Zealand origin; Figs 2A and 3A, Tables 1 and S1). Each of these stowaways had a unique haplotype, with an inferred Australian origin between Brisbane and Sydney (Figs 2A and 3A; Tables 1 and S1). These haplotypes span four different native range clades (Figs 2A and 3A) and are genetically divergent (1.8–8.3%) from the haplotypes present in the established New Zealand range of *L. delicata*. Six different source regions were identified: Sydney (*n* = 4), Wyong NSW (*n* = 1), Port Macquarie NSW (*n* = 1), Gold Coast (Lamington NP, *n* = 3) and Brisbane (southern suburbs, *n* = 2; northern suburbs, *n* = 2) (Figs 2A and 3A; Table 1). Just over half (7 of 13, 53.8%) of these source regions were correctly predicted in the MPI database (note for LDN47 that Caboolture borders the northern suburbs of Brisbane) (Fig 2A, Table 1). For the source regions that did not match predictions, the inferred origin was within 100 km for three interceptions (LDN07 and LDN62: Gold Coast approximately 80 km from Brisbane; LDN23: Wyong approximately 90 km from Sydney), and over 350 km for the other two (LDN40: Sydney approximately 900 km from Brisbane; LDN231: Port Macquarie approximately 385 km from Sydney) (Fig. 2A, Table 1). The *L. delicata* stowaways from Australia were intercepted in both the North Island (Auckland, Tauranga, Wellington) and South Island (Christchurch, Invercargill), with more than half of the detections (54%) of new arrivals being in locations outside the established New Zealand range (Fig. 1; Tables 1 and S1).

**Table 1:** Details of the *Lampropholis delicata* specimens intercepted entering New Zealand direct from Australia. The inferred Australian origin of each specimen from the molecular data (Fig. 3AAA) is compared with the predicted origin recorded in the Ministry for Primary Industries interception database. All intercepted lizards were found alone rather than in groups

	Interception					Lizard			Origin		
				
Sample code	Location	Month	Transport method	Border or post-border	Cargo type	Snout-vent length (mm)	Adult?	Alive?	Predicted	Confirmed	Haplotype
LDN01	Wellington	October	Air	Post-border	Personal effects	28	N	Alive	Sydney	Sydney	MAF01
LDN06	Auckland	March	Sea	Post-border	Shipping container (cosmetics, food)	30	N	Alive	Sydney	Sydney	MAF05
LDN07	Auckland	June	Air	Border	Personal effects	39	Y	Alive	Brisbane	Gold Coast-Lamington NP	MAF06
LDN23	Auckland	June	Sea	Post-border	Shipping container (light fittings)	30	N	Alive	North Sydney (Brookvale)	North NSW coast-Wyong	MAF13
LDN26	Wellington	August	Sea	Post-border	Shipping container (household effects)	34	N	Alive	Gold Coast	Gold Coast-Lamington NP	MAF16
LDN40	Auckland	October	Air	Border	Personal effects	35	Y	Alive	Sydney?	Brisbane (South)	MAF17
LDN47	Tauranga	April	Air	Post-border	Personal effects	26	N	Alive	Caboolture	Brisbane (North)	MAF18
LDN57	Christchurch	April	Air	Post-border	Personal effects	32	N	Alive	Brisbane	Brisbane (South)	MAF19
LDN62	Christchurch	August	Sea	Border	Shipping container (household effects)	40	Y	Dead	Brisbane (Kingston)	Gold Coast-Lamington NP	MAF20
LDN64	Christchurch	October	Sea	Border	Shipping container (household effects)	37	Y	Alive	Brisbane (Samford)	Brisbane (North)	MAF21
LDN218	Auckland	December	Air	Post-border	Personal effects	36	Y	Alive	Unknown	Sydney	MAF10
LDN230	Wellington	May	Sea	Border	Shipping container (mixed freight)	36	Y	Alive	Sydney	Sydney	MAF14
LDN231	Invercargill	June	Air	Post-border	Personal effects	35	Y	Alive	Sydney	North NSW coast-Port Macquarie	MAF15

### *Lampropholis guichenoti* phylogenetic analyses

The edited *ND4* alignment comprised 671 characters, of which, 264 (39.3%) were variable and 200 (29.8%) were parsimony informative. For the ingroup only, the alignment contained 197 (29.4%) variable characters, of which, 166 (24.7%) were parsimony informative. Base frequencies were unequal (A = 0.326, T = 0.255, C = 0.290, G = 0.129), but a chi-square test confirmed the homogeneity of base frequencies among sequences (χ^2^ = 24.69, df = 384, *P* = 1.0).

The AIC from Modeltest supported the GTR+I+G substitution model as the most appropriate for our data set. Parameters estimated under this model were as follows: relative substitution rates (A↔C = 2.0546, A↔G = 63.4127, A↔T = 2.4890, C↔G = 0.5442, C↔T = 26.3913, relative to G↔T = 1.0000), proportion of invariable sites (0.4329), and gamma distribution shape parameter (0.8939). The topologies of the NJ, ML and Bayesian trees were almost identical; therefore, we present a phylogenetic tree with ML bootstrap (BS) values and Bayesian PP indicating branch support (Fig. 3A). The same two lineages and subclades identified in Chapple et al. (2011c) were recovered (Fig. 3B).

### Origin of *Lampropholis guichenoti* interceptions

All four *L. guichenoti* individuals intercepted entering New Zealand had a unique haplotype (Fig. 3B, Tables 3 and S1). The source location for each of the four stowaways was correctly predicted in the MPI database (note that Eden and Bega are both in south-eastern NSW near the border with Victoria) (Fig. 2B, Table 3). The genetic distance among the four haplotypes was 1.2–7.5%, with each occurring within the southern lineage of *L. guichenoti*, but across three different subclades (S1, S2, S6) (Figs 2B and 3B). The *L. guichenoti* individuals were intercepted at three different locations in the North Island (Auckland, Rotorua, Wellington) (Fig. 1, Tables 3 and S1).

### Analysis of biosecurity interceptions of *Lampropholis* lizards

Our genetic results enabled an analysis of the introduction pathways and stowaway characteristics of *Lampropholis* lizards arriving in New Zealand from Australia, and the mechanisms of the post-border spread in *L. delicata*. Biosecurity intercepts of *L. delicata* entering New Zealand (summer: 8%, autumn: 31%, winter: 38%, spring: 23%) and moving within the country (summer: 17%, autumn: 21%, winter: 29%, spring: 33%) were more frequent during the cooler months of the year (Tables 1 and 2). The stowaways arrived in New Zealand via both air (personal effects; *L. delicata*: 54%, *L. guichenoti*: 75%) and sea (shipping containers; *L. delicata*: 46%, *L. guichenoti*: 25%) transport vectors (Tables 3). The spread of *L. delicata* within New Zealand is mostly via rail or line-haul trucks, even when shipping containers are involved (Table 2). The new arrivals into New Zealand generally evaded the initial border checks and were only detected post-border (*L. delicata*: 62%, *L. guichenoti*: 75%) (Tables 3). Most lizards survived transportation and were alive when detected (*L. delicata* new arrivals: 92%, *L. delicata* extra-limital movements within New Zealand: 79%, *L. guichenoti*: 100%) (Tables 3). All interceptions of *Lampropholis* lizards involved a single individual, except for a shipping container arriving in Wellington from Melbourne in which approximately 8 *L. guichenoti* were found (LDN229 was the only specimen retained before the shipment was fumigated) (Table 3). Around half of all *Lampropholis* stowaways were adults (*L. delicata* new arrivals: 54%, *L. delicata* extra-limital movements within New Zealand: 58%, *L. guichenoti*: 50%) (Tables 3).

**Table 2:** Details of the *Lampropholis delicata* specimens intercepted being accidentally transported within New Zealand to regions beyond the established range. All intercepted lizards were found alone rather than in groups. Note that *L. delicata* did not establish in Palmerston North until approximately 2007

	Interception					Lizard				
				
Sample code	Location	Month	Transport method	Cargo type	Cargo contents	Snout-vent length	Adult?	Alive?	Predicted origin	Haplotype
LDN05	Palmerston North	February	Truck	Freight	Pet food	40	Y	Dead	Auckland	MAF03/NZ1
LDN11	Havelock North	August	Truck	Freight	Building materials	39	Y	Dead	Auckland	MAF02/NZ4
LDN12	Dunedin	August	Truck or trail	Freight	Timber	35	Y	Alive	Auckland	MAF07/NZ5
LDN16	Palmerston North	October	Truck	Freight	Machinery	40	Y	Alive	Unknown	MAF02/NZ4
LDN17	Christchurch	October	Truck or trail	Freight	Steel	49	Y	Alive	Auckland	MAF08/NZ3
LDN18	Christchurch	December	Truck	Freight	Decorations	28	N	Dead	Waihi Beach	MAF03/NZ1
LDN22	Christchurch	May	Rail	Container	Mixed freight	33	N	Alive	Auckland	MAF04/NZ6
LDN24	Nelson	June	Sea	Shipping container	Household effects	44	Y	Alive	Auckland	MAF09/NZ2
LDN29	Palmerston North	October	Truck	Courier	Computer	37	Y	Alive	Auckland	MAF03/NZ1
LDN30	Palmerston North	October	Truck	Freight	Engine parts	30	N	Alive	Auckland	MAF03/NZ1
LDN33	Christchurch	May	Sea	Shipping container	Beverages	40	Y	Alive	Auckland	MAF03/NZ1
LDN37	Christchurch	July	Truck	Freight	Electrical fittings	39	Y	Alive	Auckland	MAF03/NZ1
LDN45	Palmerston North	February	Truck	Freight	Building materials	38	Y	Alive	Auckland	MAF08/NZ3
LDN49	Palmerston North	May	Truck	Freight	Pipe fittings	36	Y	Alive	Auckland	MAF08/NZ3
LDN51	Porirua	July	Truck	Freight	Vegetables	22	N	Alive	Auckland	MAF03/NZ1
LDN58	New Plymouth	May	Truck	Freight	New car	37	Y	Alive	Auckland	MAF03/NZ1
LDN61	Christchurch	June	Rail or sea	Container	Unknown	38	Y	Alive	Auckland	MAF03/NZ1
LDN68	Napier	March	Truck	Freight	Plasticware	14	N	Dead	Auckland	MAF09/NZ2
LDN217	Dunedin	November	Truck	Freight	Mail	30	N	Alive	Unknown	MAF03/NZ1
LDN219	Napier	December	Truck	Freight	Mail	27	N	Alive	Auckland	MAF11
LDN223	Palmerston North	October	Unknown	Unknown	Unknown	34	N	Alive	Unknown	MAF08/NZ3
LDN224	Stratford	October	Truck	Freight	Ceramics	26	N	Alive	Auckland	MAF08/NZ3
LDN225	Rotorua	November	Truck	Freight	Beverages	39	Y	Dead	Auckland	MAF12
LDN232	New Plymouth	July	Truck	Freight	Steel	40	Y	Alive	Auckland	MAF03/NZ1

**Table 3:** Details of the *Lampropholis guichenoti* specimens intercepted entering New Zealand. The inferred Australian origin of each specimen from the molecular data (Fig. 3B) is compared with the predicted origin recorded in the Ministry for Primary Industries interception database. All intercepted lizards were found alone, except for LDN229 that was one of eight individuals found in the same shipping container

	Interception					Lizard			Origin		
				
Sample code	Location	Month	Transport method	Border or post-border	Cargo type	Snout-vent length	Adult?	Alive?	Predicted	Confirmed	Haplotype
LDN65	Auckland/Rotorua	April	Air	Post-border	Personal effects	32	N	Alive	Bega, NSW	NSW/VIC border region	GNZ1
LDN66	Auckland	May	Sea	Post-border	Shipping container (ceramics)	42	Y	Alive	Adelaide	Adelaide	GNZ2
LDN67	Auckland/Rotorua	December	Sea	Post-border	Shipping container (household effects)	44	Y	Alive	Eden, NSW	NSW/VIC border region	GNZ3
LDN229	Wellington	February	Sea	Border	Shipping container	18	N	Alive	Adelaide or Melbourne	Melbourne region	GNZ4

## Discussion

### Geographic origin and transport pathways of *Lampropholis* stowaways

Our molecular analysis of the *L. delicata* specimens intercepted by MPI between 2001 and 2008 confirmed that 13 stowaways represented new arrivals direct from Australia. Genetic analysis of invertebrate species intercepted entering New Zealand have previously been used to confirm species identification (Armstrong and Ball 2005; Ball and Armstrong 2006) or their country of origin (Corin et al. 2007, 2008), but none have been able to accurately pinpoint the source region. Our ability to identify six source regions in eastern Australia for the *L. delicata* hitchhikers entering New Zealand greatly enhances our knowledge of the introduction pathways and transport vectors that are important for the human-mediated spread of the species. Most (81%) of the interceptions predicted by MPI to represent human-mediated transport of *L. delicata* from Australia to New Zealand were confirmed as new arrivals by our molecular data. Based on the import pathway data, the source region was correctly identified in the MPI database for more than half of these interceptions (54%), with the broad region of origin accurately predicted for several others (23%). MPI correctly identified the origin of all the *L. guichenoti* interceptions. While there remains scope to improve the data collected by MPI for the herpetofauna interception database, it provides a valuable resource for assessing the effectiveness of existing biosecurity measures.

Although mtDNA data alone will not be capable of distinguishing between new arrivals from the same Australian source region (Tenterfield, northern NSW) and haplotypes in the established New Zealand populations, our integration of a phylogeographic approach and an analysis of the MPI lizard interception database enhances our ability to detect such additional introductions. We demonstrate that the MPI interception database accurately (81% success rate) predicted whether *L. delicata* detections represented new arrivals from Australia, and was able to pinpoint (77% success rate) their region of origin within the native Australian range. It is unlikely that the three incorrect new arrival predictions in the current study (Table S1) represent introductions from the same source region, as they were detected in freight that originated in Melbourne (approximately 1500 km from Tenterfield). Thus, using the MPI interception database in conjunction with the mtDNA data decreases the potential for incorrectly categorizing the type of detection that each intercepted *L. delicata* represents.

The source regions for *L. delicata* stowaways include two major transport hubs in eastern Australia (Brisbane, Sydney), and multiple detections originating from each of these regions. Importantly, the five source regions for the successful introductions of *L. delicata* to Lord Howe Island and the Hawaiian Islands are also located between Brisbane and Sydney (Chapple et al. 2012c), indicating biosecurity agencies may need to prioritize the screening of freight and cargo from this region as they pose a higher risk for *L. delicata* introductions. In contrast, the three identified source regions for the *L. guichenoti* interceptions in New Zealand (southern NSW, Melbourne, Adelaide) are all at higher latitudes than for the *L. delicata* stowaways. Although the human-mediated transportation of *L. guichenoti* appears to be infrequent, the source regions in south-eastern Australia have high climatic similarity to New Zealand (Peacock and Worner 2006), which might increase the likelihood of successful establishment in New Zealand in the future.

*Lampropholis* skinks used both air (personal effects) and sea (shipping containers) transport routes to reach New Zealand, and although intercepted at transport hubs in both the North and South Islands, Auckland was the port of entry for half the stowaways. Our analyses highlight two significant concerns: (i) new *L. delicata* stowaways from Australia regularly arrive in regions beyond the established range in New Zealand, and (ii) most (65%) of *Lampropholis* arrivals from Australia evade detection during border checks. Despite New Zealand having a comprehensive approach to biosecurity (Meyerson and Reaser 2002; Toy and Newfield 2010), previous studies of ants (Ward et al. 2006) and lizards (Gill et al. 2001) have shown that a substantial proportion of stowaways are detected post-border by biosecurity agencies. This acts to emphasize the importance of post-border management strategies (e.g. early detection, control/mitigation programs) in preventing the establishment and spread of invasive species. If new arrivals from Australia manage to successfully establish in New Zealand, *L. delicata* may be able to extend their distribution within the country. The *L. delicata* stowaways have the potential to introduce additional genetic variation into the New Zealand population, as hitchhikers were genetically divergent (1.8–8.3%) from the established population. Admixture amongst individuals from geographically distinct source regions may enhance the species capacity to adapt to challenging environmental conditions (e.g. Kolbe et al. 2004; Chapple et al. 2012c) and expand into regions of the central North Island and South Island that were previously thought to be unsuitable.

### *Lampropholis delicata* is spreading within New Zealand via human-mediated ‘jump’ dispersal

The post-border spread of nonnative species has generally received less attention than efforts to prevent or detect their initial arrival into a country (Forrest et al. 2009). Accurate movement pathway data and our molecular analysis of *L. delicata* in the MPI data set provide a detailed demonstration of how an invasive species can spread across the landscape once it establishes in a new region. More than a third (39%) of interceptions by MPI represented human-mediated movements of *L. delicata* to locations outside of their established range. In addition to the same air (freight, mail) and sea (shipping containers) transport vectors that were responsible for the species initial arrival in New Zealand, this post-border spread of *L. delicata* by human-mediated ‘jump dispersal’ also involved long-distance truck and rail transport. Jump dispersal has facilitated the rapid spread of several other invasive species, including Argentine ants (*Linepithema humile*, Suarez et al. 2001), fire ants (*Solenopsis invicta*, King et al. 2009), land snails (*Xeropicta derbentina*, Aubry et al. 2006) and cane toads (*Bufo marinus*, White and Shine 2009) (reviewed in Wilson et al. 2009; Phillips and Suarez 2012). Similar to the Argentine ant in New Zealand (Ward et al. 2005), human-mediated jump dispersal appears to be more important than natural dispersal in the rapid spread of *L. delicata* across the country.

The post-border spread of *L. delicata* in New Zealand appears to be driven by stowaways in freight originating from the Auckland region. Auckland is New Zealand's major transport hub, with half of the country's population living in the region, the bulk of the international passengers, freight and cargo transiting through the area, and most large companies operating nation-wide distribution centres within the city. It is also the region where *L. delicata* was first detected in the country, and is currently where the species is most abundant and widespread (Lever 2003; Chapple et al. 2012c). Thus, the Auckland region represents an ‘invasion hub’ (e.g. Florance et al. 2011) for *L. delicata*. Importantly, *L. delicata* is regularly being transported to regions that bioclimatic modelling suggests the species can establish (e.g. Napier, lower North Island, Nelson, Christchurch) (Peace 2004). Indeed, the frequent arrival of individuals in Palmerston North may have contributed to the recent establishment (approximately 2007) of the species in the city (A. H. Whitaker, unpublished data). Owing to a legislative quirk in the New Zealand *Wildlife Act 1953*, *L. delicata* was a protected species in New Zealand from when it initially established in the country in the early 1960s until 2010 when it was included in Schedule 5 (‘wildlife not protected’) of the *Wildlife Act* and simultaneously listed as an ‘unwanted organism’ under the *Biosecurity Act 1993*. This delay in instituting the appropriate legal status for the species may have contributed to the spread of *L. delicata* in New Zealand.

### Why is *Lampropholis delicata* so adept at human-mediated dispersal?

*Lampropholis delicata* is the only Australian lizard species that is a successful invader overseas, and the only lizard species that has become invasive in New Zealand (Lever 2003; Kraus 2009). Our analysis of the MPI interception records emphasizes the species’ remarkable propensity for human-assisted dispersal, and suggests that the resultant high propagule pressure (e.g. Lockwood et al. 2005; Simberloff 2009) enables *L. delicata* to establish in regions outside its native range. *Lampropholis delicata* has successfully established beyond its native Australian range on at least seven occasions (Lord Howe Island, five times; New Zealand, once; Hawaiian Islands, once), and on numerous occasions within New Zealand (this study) and the Hawaiian Islands (all major islands; Chapple et al. 2012c). However, despite its regular ‘jump dispersal’ to new regions, including New Zealand, *L. guichenoti* has never successfully established outside its native range (Gill et al. 2001; Kraus 2009; this study).

Successful invaders possess a range of behavioural traits (e.g. exploratory behaviour, aggression, tolerance of human-inhabited environments) that enhance their ability to make the transition through successive stages of the introduction process (Chapple et al. 2012a,b). Although the two *Lampropholis* species occur in sympatry throughout most of south-eastern Australia, *L. delicata* exhibits higher levels of exploratory behaviour that may increase its probability of getting into freight and cargo, and a greater tendency to hide in shelter, which might decrease its chances of being detected during biosecurity checks at the border (Chapple et al. 2011a; Cromie and Chapple 2012). Their exploratory behaviour may explain the more frequent transportation of *L. delicata* from Australia during the study period (13 interceptions vs 4 for *L. guichenoti*), a trend that has continued since 2009 (18 new *L. delicata* interceptions versus 1 for *L. guichenoti*; MPI unpublished data to May 2012). In the same period (2009–2012) there have been a further 13 detections of jump dispersal within New Zealand to locations beyond its established range, 82% of them from Auckland. The human-assisted dispersal of *Lampropholis* skinks is more frequent in the cooler months of the year, when individuals may be seeking warmth and shelter. Many freight and cargo items (e.g. timber, plant material, household items) provide ideal conditions for lizards to shelter, and may explain why the vast majority of *Lampropholis* stowaways were able to survive transit and arrive alive in New Zealand.

### Enhancing biosecurity protocols to prevent further lizard introductions

Anecdotal evidence suggests that *L. delicata* could adversely impact the native biota in its introduced regions (Baker 1979; Lever 2003; Peace 2004), but this has yet to be investigated in detail. However, several lizard species that adversely impact native fauna (e.g. Asian house gecko, mourning gecko; Petren et al. 1993; Dame and Petren 2006; Short and Petren 2008; Hoskin 2010) are frequent arrivals in New Zealand (Gill et al. 2001; Lever 2003; Kraus 2009; MPI, unpublished data). As unintentional introductions are often difficult to detect and control (Allen et al. 2006), and border checks only detect a small proportion of sheltering stowaways (Ward et al. 2006; Toy and Newfield 2010), the actual number of lizards entering New Zealand is likely much higher for many species than that documented for *L. delicata* in this study. Although species from eastern Australia may be more likely to establish in temperate New Zealand than those from the tropical regions of the Pacific (Gill et al. 2001; Ward et al. 2006), the invasive Asian house gecko is now widespread throughout south-east Queensland (Hoskin 2010), a known source region for *L. delicata* introductions (Chapple et al. 2012c; this study). The number of shipping containers, air passengers and freight/cargo inspected at New Zealand's borders has increased over the past decade (Hayden and Whyte 2003; Ward et al. 2006). Targeted screening of shipments originating from eastern Australia (particularly the Brisbane-Sydney region) and enhanced checks for sheltering lizards may help prevent the establishment of additional invasive lizard species in New Zealand.

## Conclusions

The integration of interception databases with the phylogeographic analysis of the intercepted specimens could be adopted more broadly by biosecurity agencies. Our study demonstrates that this approach is not only capable of confirming the identity of the detected individuals but can provide detailed information on introduction pathways and mechanisms of post-border spread. Importantly, the approach may indicate whether individuals from particular transport hubs have a higher likelihood of surviving transportation and evading border checks and identify particular traits (e.g. body size, life-history stage) that enhance the propensity for human-mediated dispersal. While it might not be necessary to routinely obtain DNA sequence from every interception, we recommend that the specimens should be treated (tissue sampling, specimen preservation) and stored (voucher specimen and tissue sample collections) in a manner that enables future genetic (e.g. mtDNA data) and morphological analysis of the detections.

## Data archiving statement

Data for this study are available at GenBank: EF567304, EF567726, EF567768-EU567769, HQ454789-HQ454913, JF438009-JF438483, JF915805-JF915811 and JQ413190-JQ413223.

## Supporting Information

**Table S1.** Details of the *Lampropholis delicata* and *L. guichenoti* specimens intercepted by MPI between 2001 and 2008.

**Table S2.** Locality data, museum voucher specimen information, haplotype data, and GenBank accession numbers for the samples from the native range of *Lampropholis delicata*.

**Table S3.** Locality data, museum voucher specimen information, haplotype data, and GenBank accession numbers for the samples from the established range of *Lampropholis delicata* in New Zealand.

**Table S4.** Locality data, museum voucher specimen information, haplotype data, and GenBank accession numbers for the samples from the native range of *Lampropholis guichenoti*.
