# Supplementary material for: Biosecurity interceptions of an invasive lizard: origin of stowaways and human-assisted spread within New Zealand
Source: Evol Appl. 2012 Sep 3;6(2):324–39. doi: 10.1111/eva.12002 (PMC3586621; doi:10.1111/eva.12002)
Supplement: Supplementary file 1 [file eva0006-0324-SD1.doc]

**Table S1.** Details of the *Lampropholis delicata* (LD) and *L. guichenoti* (LG) specimens intercepted by MPI between 2001 and 2008. The haplotype and GenBank accession numbers are provided for each specimen. WCL= Whitaker Consultants Ltd specimen code.

|  |  |  |  | Interception | | |  | GenBank Accession Number | |
| --- | --- | --- | --- | --- | --- | --- | --- | --- | --- |
| Species | Sample Code | WCL Code | Date | Location | Predicted Intro Mode | Confirmed Intro Mode | Haplotype | ND2 | ND4 |
| LD | LDN01 | WCL37 | Oct 2001 | Wellington airport | new arrival | new arrival | MAF01 | JQ413190 | JQ413205 |
| LD | LDN02 | WCL46 | Nov 2001 | Mangere, Auckland | local NZ | local NZ | MAF02/NZ4 | JF915794 | JF915808 |
| LD | LDN03 | WCL55 | Jan 2002 | Mt Wellington, Auckland | local NZ | local NZ | MAF03/NZ1 | JF915791 | JF915805 |
| LD | LDN04 | WCL55 | Jan 2002 | Mt Wellington, Auckland | local NZ | local NZ | MAF04/NZ6 | JF915796 | JF915810 |
| LD | LDN05 | WCL63 | Feb 2002 | Palmerston North | extralimital within NZ | extralimital within NZ | MAF03/NZ1 | JF915791 | JF915805 |
| LD | LDN06 | WCL67 | March 2002 | Auckland | new arrival | new arrival | MAF05 | JQ413191 | JQ413206 |
| LD | LDN07 | WCL79 | June 2002 | Auckland | new arrival | new arrival | MAF06 | JQ413192 | JQ413207 |
| LD | LDN08 | WCL80 | June 2002 | Glen Eden, Auckland | local NZ | local NZ | MAF03/NZ1 | JF915791 | JF915805 |
| LD | LDN09 | WCL81 | July 2002 | Onehunga, Auckland | local NZ | local NZ | MAF02/NZ4 | JF915794 | JF915808 |
| LD | LDN10 | WCL95 | Aug 2002 | Mt Maunganui, Tauranga | local NZ | local NZ | MAF03/NZ1 | JF915791 | JF915805 |
| LD | LDN11 | WCL97 | Aug 2002 | Havelock North | extralimital within NZ | extralimital within NZ | MAF02/NZ4 | JF915794 | JF915808 |
| LD | LDN12 | WCL102 | Aug 2002 | Dunedin | extralimital within NZ | extralimital within NZ | MAF07/NZ5 | JF915795 | JF915809 |
| LD | LDN13 | WCL103 | Aug 2002 | Auckland | new arrival? | local NZ | MAF03/NZ1 | JF915791 | JF915805 |
| LD | LDN14 | WCL115 | Oct 2002 | Tauranga | local NZ | local NZ | MAF03/NZ1 | JF915791 | JF915805 |
| LD | LDN15 | WCL116 | Oct 2002 | Auckland airport | local NZ | local NZ | MAF03/NZ1 | JF915791 | JF915805 |
| LD | LDN16 | WCL125 | Oct 2002 | Palmerston North | extralimital within NZ | extralimital within NZ | MAF02/NZ4 | JF915794 | JF915808 |
| LD | LDN17 | WCL127 | Oct 2002 | Christchurch | extralimital within NZ | extralimital within NZ | MAF08/NZ3 | JF915793 | JF915807 |
| LD | LDN18 | WCL141 | Dec 2002 | Avonhead, Christchurch | extralimital within NZ | extralimital within NZ | MAF03/NZ1 | JF915791 | JF915805 |
| LD | LDN19 | WCL161 | March 2003 | Mangere, Auckland | local NZ | local NZ | MAF03/NZ1 | JF915791 | JF915805 |
| LD | LDN20 | WCL167 | March 2003 | Newmarket, Auckland | local NZ | local NZ | MAF03/NZ1 | JF915791 | JF915805 |
| LD | LDN21 | WCL183 | May 2003 | Newmarket, Auckland | local NZ | local NZ | MAF09/NZ2 | JF915792 | JF915806 |
| LD | LDN22 | WCL188 | May 2003 | Sockburn, Christchurch | extralimital within NZ | extralimital within NZ | MAF04/NZ6 | JF915796 | JF915810 |
| LD | LDN23 | WCL202 | June 2003 | Avondale, Auckland | new arrival | new arrival | MAF13 | JQ413196 | JQ413211 |
| LD | LDN24 | WCL203 | June 2003 | Nelson | extralimital within NZ | extralimital within NZ | MAF09/NZ2 | JF915792 | JF915806 |
| LD | LDN25 | WCL206 | July 2003 | Mt Wellington, Auckland | local NZ | local NZ | MAF03/NZ1 | JF915791 | JF915805 |
| LD | LDN26 | WCL215 | Aug 2003 | Lower Hutt, Wellington | new arrival | new arrival | MAF16 | JQ413199 | JQ413214 |
| LD | LDN27 | WCL217 | Aug 2003 | Auckland | local NZ | local NZ | MAF03/NZ1 | JF915791 | JF915805 |
| LD | LDN28 | WCL231 | Sept 2003 | Avondale, Auckland | local NZ | local NZ | MAF03/NZ1 | JF915791 | JF915805 |
| LD | LDN29 | WCL238 | Oct 2003 | Palmerston North | extralimital within NZ | extralimital within NZ | MAF03/NZ1 | JF915791 | JF915805 |
| LD | LDN30 | WCL239 | Oct 2003 | Palmerston North | new arrival | extralimital within NZ | MAF03/NZ1 | JF915791 | JF915805 |
| LD | LDN31 | WCL255 | Nov 2003 | Manukau City, Auckland | local NZ | local NZ | MAF03/NZ1 | JF915791 | JF915805 |
| LD | LDN32 | WCL318 | April 2004 | Otahuhu, Auckland | local NZ | local NZ | MAF08/NZ3 | JF915793 | JF915807 |
| LD | LDN33 | WCL347 | May 2004 | Hornby, Christchurch | extralimital within NZ | extralimital within NZ | MAF03/NZ1 | JF915791 | JF915805 |
| LD | LDN34 | WCL351 | June 2004 | East Tamaki, Auckland | local NZ | local NZ | MAF03/NZ1 | JF915791 | JF915805 |
| LD | LDN35 | WCL352 | June 2004 | Newmarket, Auckland | local NZ | local NZ | MAF03/NZ1 | JF915791 | JF915805 |
| LD | LDN36 | WCL362 | May 2004 | Onehunga, Auckland | local NZ | local NZ | MAF03/NZ1 | JF915791 | JF915805 |
| LD | LDN37 | WCL365 | July 2004 | Christchurch | extralimital within NZ | extralimital within NZ | MAF03/NZ1 | JF915791 | JF915805 |
| LD | LDN38 | WCL372 | Aug 2004 | Mangere, Auckland | local NZ | local NZ | MAF03/NZ1 | JF915791 | JF915805 |
| LD | LDN39 | WCL384 | Sept 2004 | New Lynn, Auckland | local NZ | local NZ | MAF03/NZ1 | JF915791 | JF915805 |
| LD | LDN40 | WCL406 | Oct 2004 | Auckland airport | new arrival | new arrival | MAF17 | JQ413200 | JQ413215 |
| LD | LDN41 | WCL425 | Dec 2004 | Auckland airport | local NZ | local NZ | MAF03/NZ1 | JF915791 | JF915805 |
| LD | LDN42 | WCL428 | Jan 2005 | Auckland airport | local NZ | local NZ | MAF03/NZ1 | JF915791 | JF915805 |
| LD | LDN43 | WCL435 | Jan 2005 | Newmarket, Auckland | local NZ | local NZ | MAF03/NZ1 | JF915791 | JF915805 |
| LD | LDN44 | WCL437 | Jan 2005 | Manurewa, Auckland | local NZ | local NZ | MAF03/NZ1 | JF915791 | JF915805 |
| LD | LDN45 | WCL450 | Feb 2005 | Palmerston North | extralimital within NZ | extralimital within NZ | MAF08/NZ3 | JF915793 | JF915807 |
| LD | LDN46 | WCL466 | March 2005 | Tauranga | local NZ | local NZ | MAF03/NZ1 | JF915791 | JF915805 |
| LD | LDN47 | WCL473 | April 2005 | Auckland/Tauranga | new arrival | new arrival | MAF18 | JQ413201 | JQ413216 |
| LD | LDN48 | WCL494 | May 2005 | Manurewa, Auckland | local NZ | local NZ | MAF03/NZ1 | JF915791 | JF915805 |
| LD | LDN49 | WCL495 | May 2005 | Palmerston North | extralimital within NZ | extralimital within NZ | MAF08/NZ3 | JF915793 | JF915807 |
| LD | LDN50 | WCL506 | July 2005 | Glendene, Auckland | local NZ | local NZ | MAF03/NZ1 | JF915791 | JF915805 |
| LD | LDN51 | WCL520 | July 2005 | Porirua | extralimital within NZ | extralimital within NZ | MAF03/NZ1 | JF915791 | JF915805 |
| LD | LDN52 | WCL529 | Sept 2005 | Glendene, Auckland | local NZ | local NZ | MAF03/NZ1 | JF915791 | JF915805 |
| LD | LDN53 | WCL567 | Nov 2005 | Mangere, Auckland | new arrival | local NZ | MAF03/NZ1 | JF915791 | JF915805 |
| LD | LDN54 | WCL568 | Nov 2005 | Wiri, Auckland | local NZ | local NZ | MAF03/NZ1 | JF915791 | JF915805 |
| LD | LDN55 | WCL616 | Jan 2006 | Hamilton | local NZ | local NZ | MAF03/NZ1 | JF915791 | JF915805 |
| LD | LDN56 | WCL620 | March 2006 | Auckland | local NZ | local NZ | MAF11 | JQ413194 | JQ413209 |
| LD | LDN57 | WCL622 | April 2006 | Christchurch airport | new arrival | new arrival | MAF19 | JQ413202 | JQ413217 |
| LD | LDN58 | WCL637 | May 2006 | New Plymouth | extralimital within NZ | extralimital within NZ | MAF03/NZ1 | JF915791 | JF915805 |
| LD | LDN59 | WCL642 | May 2006 | Auckland airport | local NZ | local NZ | MAF03/NZ1 | JF915791 | JF915805 |
| LD | LDN60 | WCL668 | July 2006 | Auckland airport | new arrival? | local NZ | MAF03/NZ1 | JF915791 | JF915805 |
| LD | LDN61 | WCL671 | June 2006 | Sockburn, Christchurch | extralimital within NZ | extralimital within NZ | MAF03/NZ1 | JF915791 | JF915805 |
| LD | LDN62 | WCL672 | Aug 2006 | Hornby, Christchurch | new arrival | new arrival | MAF20 | JQ413203 | JQ413218 |
| LD | LDN63 | WCL699 | Aug 2006 | Auckland | local NZ | local NZ | MAF09/NZ2 | JF915792 | JF915806 |
| LD | LDN64 | WCL714 | Oct 2006 | Avonside, Christchurch | new arrival | new arrival | MAF21 | JQ413204 | JQ413219 |
| LD | LDN68 | WCL165 | March 2003 | Napier | new arrival | extralimital within NZ | MAF09/NZ2 | JF915792 | JF915806 |
| LD | LDN215 | WCL816 | Dec 2007 | Sandringham, Auckland | new arrival? | within NZ | MAF03/NZ1 | JF915791 | JF915805 |
| LD | LDN216 | WCL726 | Nov 2006 | Auckland airport | local NZ | local NZ | MAF03/NZ1 | JF915791 | JF915805 |
| LD | LDN217 | WCL728 | Nov 2006 | Dunedin | new arrival? | extralimital within NZ | MAF03/NZ1 | JF915791 | JF915805 |
| LD | LDN218 | WCL732 | Dec 2006 | Auckland airport | new arrival | new arrival | MAF10 | JQ413193 | JQ413208 |
| LD | LDN219 | WCL740 | Dec 2006 | Napier | extralimital within NZ | extralimital within NZ | MAF11 | JQ413194 | JQ413209 |
| LD | LDN220 | WCL744 | Jan 2007 | Mt Roskill, Auckland | local NZ | local NZ | MAF03/NZ1 | JF915791 | JF915805 |
| LD | LDN221 | WCL754 | Feb 2007 | Manurewa, Auckland | local NZ | local NZ | MAF03/NZ1 | JF915791 | JF915805 |
| LD | LDN222 | WCL761 | April 2007 | Auckland | local NZ | local NZ | MAF03/NZ1 | JF915791 | JF915805 |
| LD | LDN223 | WCL795 | Oct 2007 | Palmerston North | extralimital within NZ | extralimital within NZ | MAF08/NZ3 | JF915793 | JF915807 |
| LD | LDN224 | WCL800 | Oct 2007 | Stratford | extralimital within NZ | extralimital within NZ | MAF08/NZ3 | JF915793 | JF915807 |
| LD | LDN225 | WCL801 | Nov 2007 | Rotorua | extralimital within NZ | extralimital within NZ | MAF12 | JQ413195 | JQ413210 |
| LD | LDN230 | WCL830 | May 2008 | Wellington | new arrival | new arrival | MAF14 | JQ413197 | JQ413212 |
| LD | LDN231 | WCL839 | June 2008 | Invercargill | new arrival | new arrival | MAF15 | JQ413198 | JQ413213 |
| LD | LDN232 | WCL843 | July 2008 | New Plymouth | new arrival? | extralimital within NZ | MAF03/NZ1 | JF915791 | JF915805 |
| LG | LDN65 | WCL78 | April 2002 | Auckland/Rotorua | new arrival | new arrival | GNZ1 | — | JQ413220 |
| LG | LDN66 | WCL92 | May 2002 | Ponsonby, Auckland | new arrival | new arrival | GNZ2 | — | JQ413223 |
| LG | LDN67 | WCL584 | Dec 2005 | Auckland/Rotorua | new arrival | new arrival | GNZ3 | — | JQ413221 |
| LG | LDN229 | WCL819 | Feb 2008 | Wellington | new arrival | new arrival | GNZ4 | — | JQ413222 |
