# Supplementary material for: Biosecurity interceptions of an invasive lizard: origin of stowaways and human-assisted spread within New Zealand
Source: Evol Appl. 2012 Sep 3;6(2):324–39. doi: 10.1111/eva.12002 (PMC3586621; doi:10.1111/eva.12002)
Supplement: Supplementary file 3 [file eva0006-0324-SD3.doc]

**Table S3.** Locality data, museum voucher specimen information, and GenBank accession numbers for the samples used in this study from the established range of *Lampropholis delicata* in New Zealand. Museum abbreviation: RE = National Museum of New Zealand, Te Papa Tongarewa, Wellington, New Zealand.

| Pop. | Collection Locality | Sample  Code | Museum Voucher | Tissue Code | Haplotype | GenBank Accession Numbers | |
| --- | --- | --- | --- | --- | --- | --- | --- |
|  |  |  |  |  |  | ND2 | ND4 |
| N1 | Lake Ngatu, Kaitaia, Northland, New Zealand | LDN226 |  |  | NZ4 | JF915794 | JF915808 |
|  |  | LDN227 |  |  | NZ4 | JF915794 | JF915808 |
|  |  | LDN228 |  |  | NZ4 | JF915794 | JF915808 |
| N2 | Department of Conservation Area office, Whangarei, Northland, New Zealand | LDN162 |  |  | NZ6 | JF915796 | JF915810 |
|  |  | LDN163 | RE 005865 |  | NZ1 | JF915791 | JF915805 |
|  |  | LDN164 | RE 005866 |  | NZ1 | JF915791 | JF915805 |
|  |  | LDN165 | RE 005867 |  | NZ1 | JF915791 | JF915805 |
| N3 | Dargaville, Northland, New Zealand | LDN166 | RE 005868 |  | NZ1 | JF915791 | JF915805 |
|  |  | LDN167 | RE 005869 |  | NZ1 | JF915791 | JF915805 |
|  |  | LDN168 | RE 005870 |  | NZ1 | JF915791 | JF915805 |
|  |  | LDN169 | RE 005871 |  | NZ1 | JF915791 | JF915805 |
|  |  | LDN170 | RE 005872 |  | NZ1 | JF915791 | JF915805 |
|  |  | LDN171 | RE 005873 |  | NZ1 | JF915791 | JF915805 |
|  |  | LDN172 | RE 005874 |  | NZ1 | JF915791 | JF915805 |
|  |  | LDN173 | RE 005875 |  | NZ1 | JF915791 | JF915805 |
|  |  | LDN174 | RE 005876 |  | NZ1 | JF915791 | JF915805 |
|  |  | LDN175 | RE 005877 |  | NZ1 | JF915791 | JF915805 |
|  |  | LDN176 | RE 005878 |  | NZ1 | JF915791 | JF915805 |
|  |  | LDN177 | RE 005879 |  | NZ1 | JF915791 | JF915805 |
|  |  | LDN178 | RE 005880 |  | NZ4 | JF915794 | JF915808 |
|  |  | LDN179 | RE 005881 |  | NZ1 | JF915791 | JF915805 |
| N4 | Harbour View Rd, Leigh, Northland, New Zealand | LDN104 | RE 005810 |  | NZ1 | JF915791 | JF915805 |
|  |  | LDN105 | RE 005811 |  | NZ1 | JF915791 | JF915805 |
|  |  | LDN106 | RE 005812 |  | NZ1 | JF915791 | JF915805 |
|  |  | LDN107 | RE 005813 |  | NZ1 | JF915791 | JF915805 |
|  |  | LDN108 | RE 005814 |  | NZ1 | JF915791 | JF915805 |
|  |  | LDN109 | RE 005815 |  | NZ1 | JF915791 | JF915805 |
|  |  | LDN110 | RE 005816 |  | NZ1 | JF915791 | JF915805 |
|  |  | LDN111 | RE 005817 |  | NZ1 | JF915791 | JF915805 |
|  |  | LDN112 | RE 005818 |  | NZ1 | JF915791 | JF915805 |
|  |  | LDN113 | RE 005819 |  | NZ1 | JF915791 | JF915805 |
|  |  | LDN114 | RE 005820 |  | NZ1 | JF915791 | JF915805 |
|  |  | LDN115 | RE 005821 |  | NZ1 | JF915791 | JF915805 |
|  |  | LDN116 | RE 005822 |  | NZ1 | JF915791 | JF915805 |
|  |  | LDN117 | RE 005823 |  | NZ1 | JF915791 | JF915805 |
|  |  | LDN118 | RE 005824 |  | NZ1 | JF915791 | JF915805 |
| N5 | Army Bay, Whangaparaoa Peninsula, New Zealand | LDN154 | RE 005857 |  | NZ5 | JF915795 | JF915809 |
|  |  | LDN155 | RE 005858 |  | NZ5 | JF915795 | JF915809 |
|  |  | LDN156 | RE 005859 |  | NZ5 | JF915795 | JF915809 |
|  |  | LDN158 | RE 005861 |  | NZ5 | JF915795 | JF915809 |
|  |  | LDN159 | RE 005862 |  | NZ5 | JF915795 | JF915809 |
|  |  | LDN160 | RE 005863 |  | NZ5 | JF915795 | JF915809 |
|  |  | LDN161 | RE 005864 |  | NZ5 | JF915795 | JF915809 |
| N6 | Albany Heights Rd, Albany, Auckland, New Zealand | LDN83 | RE 005789 |  | NZ1 | JF915791 | JF915805 |
|  |  | LDN84 | RE 005790 |  | NZ1 | JF915791 | JF915805 |
|  |  | LDN85 | RE 005791 |  | NZ1 | JF915791 | JF915805 |
|  |  | LDN86 | RE 005792 |  | NZ1 | JF915791 | JF915805 |
|  |  | LDN87 | RE 005793 |  | NZ1 | JF915791 | JF915805 |
|  |  | LDN88 | RE 005794 |  | NZ1 | JF915791 | JF915805 |
|  |  | LDN89 | RE 005795 |  | NZ1 | JF915791 | JF915805 |
|  |  | LDN90 | RE 005796 |  | NZ1 | JF915791 | JF915805 |
|  |  | LDN91 | RE 005797 |  | NZ1 | JF915791 | JF915805 |
|  |  | LDN92 | RE 005798 |  | NZ1 | JF915791 | JF915805 |
|  |  | LDN93 | RE 005799 |  | NZ1 | JF915791 | JF915805 |
|  |  | LDN94 | RE 005800 |  | NZ1 | JF915791 | JF915805 |
|  |  | LDN95 | RE 005801 |  | NZ1 | JF915791 | JF915805 |
|  |  | LDN96 | RE 005802 |  | NZ1 | JF915791 | JF915805 |
|  |  | LDN97 | RE 005803 |  | NZ1 | JF915791 | JF915805 |
|  |  | LDN98 | RE 005804 |  | NZ1 | JF915791 | JF915805 |
|  |  | LDN99 | RE 005805 |  | NZ1 | JF915791 | JF915805 |
|  |  | LDN100 | RE 005806 |  | NZ1 | JF915791 | JF915805 |
|  |  | LDN101 | RE 005807 |  | NZ1 | JF915791 | JF915805 |
|  |  | LDN102 | RE 005808 |  | NZ1 | JF915791 | JF915805 |
|  |  | LDN103 | RE 005809 |  | NZ1 | JF915791 | JF915805 |
| N7 | Rosebank Rd, Avondale, Auckland, New Zealand | LDN119 | RE 005825 |  | NZ2 | JF915792 | JF915806 |
|  |  | LDN120 | RE 005826 |  | NZ2 | JF915792 | JF915806 |
|  |  | LDN121 | RE 005827 |  | NZ1 | JF915791 | JF915805 |
|  |  | LDN122 | RE 005828 |  | NZ1 | JF915791 | JF915805 |
|  |  | LDN123 | RE 005829 |  | NZ1 | JF915791 | JF915805 |
|  |  | LDN124 | RE 005830 |  | NZ1 | JF915791 | JF915805 |
|  |  | LDN125 | RE 005831 |  | NZ2 | JF915792 | JF915806 |
|  |  | LDN126 | RE 005832 |  | NZ2 | JF915792 | JF915806 |
|  |  | LDN127 | RE 005833 |  | NZ2 | JF915792 | JF915806 |
|  |  | LDN128 | RE 005834 |  | NZ1 | JF915791 | JF915805 |
|  |  | LDN129 | RE 005835 |  | NZ1 | JF915791 | JF915805 |
|  |  | LDN130 | RE 005836 |  | NZ1 | JF915791 | JF915805 |
|  |  | LDN131 | RE 005837 |  | NZ1 | JF915791 | JF915805 |
|  |  | LDN132 | RE 005838 |  | NZ1 | JF915791 | JF915805 |
|  |  | LDN133 | RE 005839 |  | NZ1 | JF915791 | JF915805 |
|  |  | LDN134 | RE 005840 |  | NZ1 | JF915791 | JF915805 |
| N8 | Bell Rd, Otahuhu, Auckland, New Zealand | LDN144 | RE 005849 |  | NZ1 | JF915791 | JF915805 |
|  |  | LDN145 |  |  | NZ1 | JF915791 | JF915805 |
|  |  | LDN146 | RE 005850 |  | NZ1 | JF915791 | JF915805 |
|  |  | LDN148 | RE 005851 |  | NZ1 | JF915791 | JF915805 |
|  |  | LDN149 | RE 005852 |  | NZ1 | JF915791 | JF915805 |
|  |  | LDN150 | RE 005853 |  | NZ1 | JF915791 | JF915805 |
|  |  | LDN151 | RE 005854 |  | NZ4 | JF915794 | JF915808 |
|  |  | LDN152 | RE 005855 |  | NZ2 | JF915792 | JF915806 |
|  |  | LDN153 | RE 005856 |  | NZ1 | JF915791 | JF915805 |
| N9 | Otara, Auckland, New Zealand | LDN135 |  |  | NZ3 | JF915793 | JF915807 |
|  |  | LDN136 | RE 005841 |  | NZ1 | JF915791 | JF915805 |
|  |  | LDN137 | RE 005842 |  | NZ1 | JF915791 | JF915805 |
|  |  | LDN138 | RE 005843 |  | NZ1 | JF915791 | JF915805 |
|  |  | LDN139 | RE 005844 |  | NZ1 | JF915791 | JF915805 |
|  |  | LDN140 | RE 005845 |  | NZ1 | JF915791 | JF915805 |
|  |  | LDN141 | RE 005846 |  | NZ1 | JF915791 | JF915805 |
|  |  | LDN142 | RE 005847 |  | NZ1 | JF915791 | JF915805 |
|  |  | LDN143 | RE 005848 |  | NZ1 | JF915791 | JF915805 |
| N10 | Waikawau Bay, Coromandel Peninsula, New Zealand | LDN180 | RE 005882 |  | NZ1 | JF915791 | JF915805 |
|  |  | LDN181 | RE 005883 |  | NZ1 | JF915791 | JF915805 |
|  |  | LDN182 | RE 005884 |  | NZ1 | JF915791 | JF915805 |
|  |  | LDN183 | RE 005885 |  | NZ1 | JF915791 | JF915805 |
|  |  | LDN184 | RE 005886 |  | NZ1 | JF915791 | JF915805 |
|  |  | LDN185 | RE 005887 |  | NZ1 | JF915791 | JF915805 |
|  |  | LDN186 | RE 005888 |  | NZ1 | JF915791 | JF915805 |
|  |  | LDN187 | RE 005889 |  | NZ1 | JF915791 | JF915805 |
| N11 | Norton Rd, Hamilton, Waikato, New Zealand | LDN69 | RE 005775 |  | NZ1 | JF915791 | JF915805 |
|  |  | LDN70 | RE 005776 |  | NZ1 | JF915791 | JF915805 |
|  |  | LDN71 | RE 005777 |  | NZ1 | JF915791 | JF915805 |
|  |  | LDN72 | RE 005778 |  | NZ1 | JF915791 | JF915805 |
|  |  | LDN73 | RE 005779 |  | NZ1 | JF915791 | JF915805 |
|  |  | LDN74 | RE 005780 |  | NZ1 | JF915791 | JF915805 |
|  |  | LDN75 | RE 005781 |  | NZ1 | JF915791 | JF915805 |
|  |  | LDN76 | RE 005782 |  | NZ1 | JF915791 | JF915805 |
|  |  | LDN77 | RE 005783 |  | NZ1 | JF915791 | JF915805 |
|  |  | LDN78 | RE 005784 |  | NZ1 | JF915791 | JF915805 |
|  |  | LDN79 | RE 005785 |  | NZ1 | JF915791 | JF915805 |
|  |  | LDN80 | RE 005786 |  | NZ1 | JF915791 | JF915805 |
|  |  | LDN81 | RE 005787 |  | NZ1 | JF915791 | JF915805 |
|  |  | LDN82 | RE 005788 |  | NZ1 | JF915791 | JF915805 |
| N12 | Bellfield Rd, Tauranga, Bay of Plenty, New Zealand | LDN188 |  |  | NZ1 | JF915791 | JF915805 |
|  |  | LDN189 | RE 005890 |  | NZ1 | JF915791 | JF915805 |
|  |  | LDN190 | RE 005891 |  | NZ1 | JF915791 | JF915805 |
|  |  | LDN191 | RE 005892 |  | NZ1 | JF915791 | JF915805 |
|  |  | LDN192 | RE 005893 |  | NZ1 | JF915791 | JF915805 |
|  |  | LDN193 | RE 005894 |  | NZ1 | JF915791 | JF915805 |
|  |  | LDN194 | RE 005895 |  | NZ1 | JF915791 | JF915805 |
|  |  | LDN195 | RE 005896 |  | NZ1 | JF915791 | JF915805 |
|  |  | LDN196 | RE 005897 |  | NZ1 | JF915791 | JF915805 |
|  |  | LDN197 | RE 005898 |  | NZ1 | JF915791 | JF915805 |
|  |  | LDN198 | RE 005899 |  | NZ1 | JF915791 | JF915805 |
|  |  | LDN199 | RE 005900 |  | NZ1 | JF915791 | JF915805 |
|  |  | LDN200 | RE 005901 |  | NZ1 | JF915791 | JF915805 |
|  |  | LDN201 | RE 005902 |  | NZ1 | JF915791 | JF915805 |
|  |  | LDN202 | RE 005903 |  | NZ1 | JF915791 | JF915805 |
|  |  | LDN203 | RE 005904 |  | NZ1 | JF915791 | JF915805 |
|  |  | LDN204 | RE 005905 |  | NZ1 | JF915791 | JF915805 |
| N13 | Edgecumbe, Bay of Plenty, New Zealand | LDN205 | RE 005906 |  | NZ2 | JF915792 | JF915806 |
|  |  | LDN206 | RE 005907 |  | NZ2 | JF915792 | JF915806 |
|  |  | LDN207 | RE 005908 |  | NZ7 | JF915797 | JF915811 |
|  |  | LDN208 | RE 005909 |  | NZ2 | JF915792 | JF915806 |
|  |  | LDN209 | RE 005910 |  | NZ2 | JF915792 | JF915806 |
|  |  | LDN210 | RE 005911 |  | NZ7 | JF915797 | JF915811 |
|  |  | LDN211 | RE 005912 |  | NZ7 | JF915797 | JF915811 |
|  |  | LDN212 | RE 005913 |  | NZ2 | JF915792 | JF915806 |
|  |  | LDN213 | RE 005914 |  | NZ2 | JF915792 | JF915806 |
|  |  | LDN214 | RE 005915 |  | NZ2 | JF915792 | JF915806 |
