# Supplementary material for: Biosecurity interceptions of an invasive lizard: origin of stowaways and human-assisted spread within New Zealand
Source: Evol Appl. 2012 Sep 3;6(2):324–39. doi: 10.1111/eva.12002 (PMC3586621; doi:10.1111/eva.12002)
Supplement: Supplementary file 4 [file eva0006-0324-SD4.doc]

**Table S4.** Locality data, museum voucher specimen information, and GenBank accession numbers for samples from the range of *Lampropholis guichenoti* in eastern Australia. State abbreviations: QLD = Queensland; NSW = New South Wales; ACT = Australian Capital Territory; VIC = Victoria; SA = South Australia. Museum acronyms: ABTC = Australian Biological Tissue Collection (housed at the South Australian Museum); AM = Australian Museum, Sydney; ANWC = CSIRO Australian National Wildlife Collection, Canberra; NR = Australian Museum Frozen Tissue Collection; NMV = Museum Victoria, Melbourne; QM = Queensland Museum, Brisbane; SAM = South Australian Museum, Adelaide.

| Population | Collection locality | State | Sample Code | Museum Voucher | Tissue Code | Clade | GenBank Accession No. |
| --- | --- | --- | --- | --- | --- | --- | --- |
| 1 | 3 km W Rainbow Beach | QLD | LGU85 | QMJ46121 | ABTC03916 | N1 | HQ454901 |
| 2 | Marcoola | QLD | LGU97 | QMJ60616 | ABTC32142 | N1 | HQ454911 |
|  |  |  | LGU98 | QMJ60617 | ABTC32143 | N1 | HQ454912 |
| 3 | Forestlands State Forest, Bald Rock | NSW | LGU42 | AMR157150 | NR8020 | N3 | HQ454862 |
|  |  |  | LGU43 | AMR157150 | NR8021 | N3 | HQ454863 |
|  |  |  | LGU44 | AMR157149 | NR8026 | N3 | HQ454864 |
| 4 | 45 km E Glen Innes on Glen Innes-Grafton Rd | NSW | LGU36 | AMR157059 | NR7856 | N2 | HQ454856 |
|  |  |  | LGU37 | AMR157059 | NR7857 | N2 | HQ454857 |
|  |  |  | LGU38 | AMR157060 | NR7858 | N2 | HQ454858 |
| 5 | Red Range to Kingsgate Rd | NSW | LGU39 | AMR157123 | NR7974 | N2 | HQ454859 |
|  |  |  | LGU40 | AMR157123 | NR7975 | N2 | HQ454860 |
|  |  |  | LGU41 | AMR157124 | NR7976 | N2 | HQ454861 |
| 6 | Glenshiel Rd, N Guyra | NSW | LGU88 | SAMAR39092 | ABTC12335 | N2 | HQ454904 |
|  |  |  | LGU89 | SAMAR39093 | ABTC12336 | N2 | HQ454905 |
| 7 | Armidale | NSW | LGU92 | SAMAR34791 | ABTC16632 | N2 | HQ454908 |
|  |  |  | LGU93 | SAMAR34737 | ABTC16633 | N2 | HQ454909 |
| 8 | Sandy Creek, 6 km along Ebor-Gyura Road | NSW | LGU33 | AMR159629 | NR6334 | N2 | HQ454853 |
|  |  |  | LGU34 | AMR159629 | NR6335 | N2 | HQ454854 |
|  |  |  | LGU35 | AMR159630 | NR6336 | N2 | HQ454855 |
| 9 | Styx River State Forest, Beech Lookout | NSW | LGU17 | AMR138216 | NR721 | N2 | HQ454837 |
|  |  |  | LGU18 | AMR138217 | NR722 | N2 | HQ454838 |
| 10 | Poison Swamp Creek, on Road to Bendemeer | NSW | LGU45 | AMR152050 | NR5309 | N2 | HQ454865 |
|  |  |  | LGU46 | AMR152050 | NR5310 | N2 | HQ454866 |
|  |  |  | LGU47 | AMR152073 | NR5311 | N4 | HQ454867 |
| 11 | Werrikimbe NP | NSW | LGU71 | — | NMVZ10368 | N5 | HQ454889 |
|  |  |  | LGU72 | — | NMVZ10369 | N5 | HQ454890 |
| 12 | Cathedral Rock NP | NSW | LGU70 | — | NMVZ10367 | N2 | HQ454888 |
| 13 | Riamukka SF, Hell Hole Forest Rd | NSW | LGU31 | AMR148267 | NR3802 | N4 | HQ454851 |
| 14 | Rickabys Creek, near Londonderry | NSW | LGU23 | AMR145567 | NR2663 | S5 | HQ454843 |
|  |  |  | LGU24 | AMR145572 | NR2664 | S5 | HQ454844 |
|  |  |  | LGU25 | AMR145573 | NR2665 | S5 | HQ454845 |
| 15 | Castlereagh Waste Depot, near Penrith | NSW | LGU19 | AMR145987 | NR2634 | S5 | HQ454839 |
|  |  |  | LGU20 | AMR145989 | NR2635 | S5 | HQ454840 |
|  |  |  | LGU21 | AMR145990 | NR2636 | S5 | HQ454841 |
| 16 | University of Western Sydney, The Driftway | NSW | LGU22 | AMR145571 | NR2662 | S5 | HQ454842 |
|  |  |  | LGU26 | AMR146209 | NR2668 | S5 | HQ454846 |
|  |  |  | LGU27 | AMR146210 | NR2669 | S5 | HQ454847 |
| 17 | Homebush Bay, Cumbungi Wetland | NSW | LGU48 | AMR141004 | NR1904 | S5 | HQ454868 |
|  |  |  | LGU49 | AMR141005 | NR1905 | S5 | HQ454869 |
|  |  |  | LGU50 | AMR141006 | NR1906 | S5 | HQ454870 |
| 18 | Cronulla Sewage Treatment Plant, Kurnell Penin | NSW | LGU28 | AMR147721 | NR3338 | S5 | HQ454848 |
|  |  |  | LGU29 | AMR147722 | NR3339 | S5 | HQ454849 |
|  |  |  | LGU30 | AMR147723 | NR3340 | S5 | HQ454850 |
| 19 | Padstow | NSW | LDA120 | — | NMVZ10306 | S5 | HQ454790 |
| 20 | 4.6 km S Abercrombie River | NSW | LGU113 | SAMAR40818 | ABTC57505 | S4 | HQ454818 |
|  |  |  | LGU114 | SAMAR40819 | ABTC57506 | S4 | HQ454819 |
| 21 | 6 km N Goulbourn | NSW | LGU90 | SAMAR39155 | ABTC12406 | S4 | HQ454906 |
| 22 | Mack’s Reef Rd, N of Canberra | NSW | LGU5 | ANWC R5509 | ANWC R5509 | S4 | HQ454801 |
|  |  |  | LGU6 | ANWC R5512 | ANWC R5512 | S4 | HQ454802 |
|  |  |  | LGU7 | ANWC R5513 | ANWC R5513 | S4 | HQ454803 |
| 23 | Piccadilly Circus, Brindabella Ranges | ACT | LGU32 | AMR151780 | NR4926 | S4 | HQ454852 |
| 24 | Princes Hwy, VIC side VIC-NSW border | VIC | LGU65 | — | NMVZ10364 | S6 | HQ454885 |
| 25 | parkland near Genoa River Bridge, Genoa | VIC | LGU64 | — | NMVZ10363 | S6 | HQ454884 |
| 26 | Cape Conran | VIC | LGU68 | — | NMVZ10365 | S6 | HQ454886 |
|  |  |  | LGU69 | — | NMVZ10366 | S6 | HQ454887 |
|  |  |  | LDA214 | — | NMVZ10321 | S6 | HQ454796 |
|  |  |  | LDA215 | — | NMVZ10322 | S6 | HQ454797 |
|  |  |  | LDA216 | — | NMVZ10323 | S6 | HQ454798 |
|  |  |  | LDA218 | — | NMVZ10324 | S6 | HQ454799 |
| 27 | Sunset Peak, Cape Conran Nature Trail | VIC | LGU63 | — | NMVZ10362 | S6 | HQ454883 |
| 28 | Murrungowar Picnic Ground, Princes Hwy | VIC | LGU62 | — | NMVZ10361 | S6 | HQ454882 |
| 29 | Buchan Caves Reserve, Moon Hill Walk | VIC | LGU60 | — | NMVZ10359 | S7 | HQ454880 |
|  |  |  | LGU61 | — | NMVZ10360 | S7 | HQ454881 |
| 30 | Lyles Break, Jtn C608 &C620, Bruthen-Nowa Nowa Rd | VIC | LGU59 | — | NMVZ10358 | S7 | HQ454879 |
| 31 | Junction C608 & Duncan Rd, S Bruthen | VIC | LGU58 | — | NMVZ10357 | S3 | HQ454878 |
| 32 | Bruthen Walking Track, Parking Area | VIC | LGU57 | — | NMVZ10356 | S7 | HQ454877 |
| 33 | Princes Downfall, Great Alpine Hwy N Bruthen | VIC | LGU56 | — | NMVZ10355 | S3 | HQ454876 |
|  |  |  | LGU55 | — | NMVZ10354 | S3 | HQ454875 |
| 34 | Bairnsdale, rec centre Wallace St & Victoria St | VIC | LGU54 | — | NMVZ10353 | S3 | HQ454874 |
| 35 | Callignee Sth Rd, 1 km S jtn with Chester Pk Rd | VIC | LGU53 | — | NMVZ10352 | S3 | HQ454873 |
| 36 | Lake Glenmaggie, opp. Glenmaggie Cemetery | VIC | LGU52 | — | NMVZ10351 | S3 | HQ454872 |
| 37 | Drouin Nature Reserve, Pryor Rd, Drouin | VIC | LGU51 | — | NMVZ10350 | S1 | HQ454871 |
| 38 | Buckleys NR Balnarring, Mornington Peninsula | VIC | LGU16 | — | NMVZ10349 | S1 | HQ454836 |
| 39 | Lilydale Lake, Melbourne | VIC | LDA181 | NMVD73633 | NMVZ6231 | S1 | HQ454793 |
|  |  |  | LDA182 | NMVD73634 | NMVZ6232 | S1 | HQ454794 |
|  |  |  | LDA183 | NMVD73635 | NMVZ6233 | S1 | HQ454795 |
| 40 | Main Yarra Trail, Yarra Flats Park, Ivanhoe, Melbourne | VIC | LDA180 | NMVD73632 | NMVZ6230 | S1 | HQ454792 |
| 41 | Eltham | VIC | LGU94 | SAMAR35538 | ABTC23333 | S1 | HQ454910 |
| 42 | Mt. Disappointment | VIC | LGU86 | NMVD62027 | ABTC04076 | S1 | HQ454902 |
|  |  |  | LGU87 | NMVD62028 | ABTC04077 | S1 | HQ454903 |
| 43 | Lambing Gully Rd, Avenel | VIC | LGU8 | — | — | S1 | HQ454804 |
|  |  |  | LGU11 | — | — | S1 | HQ454814 |
| 44 | Alexandersons Rd (& 1 km S), Locksley | VIC | LGU12 | — | — | S1 | HQ454825 |
|  |  |  | LGU13 | — | — | S1 | HQ454834 |
|  |  |  | LGU15 | — | — | S1 | HQ454835 |
| 45 | 5 km W Stonyford | VIC | LGU110 | NMVD60948 | ABTC40851 | S1 | HQ454815 |
| 46 | Harrow-Balmoral Rd, southern Grampians | VIC | LGU74 | — | NMVZ10370 | S1 | HQ454891 |
|  |  |  | LGU75 | — | NMVZ10371 | S1 | HQ454892 |
|  |  |  | LGU76 | — | NMVZ10372 | S1 | HQ454893 |
| 47 | North Boundary Rd, Dunkeld, sthn Grampians | VIC | LGU77 | — | NMVZ10373 | S1 | HQ454894 |
|  |  |  | LGU78 | — | NMVZ10374 | S1 | HQ454895 |
|  |  |  | LGU79 | — | NMVZ10375 | S1 | HQ454896 |
|  |  |  | LGU80 | — | NMVZ10376 | S1 | HQ454897 |
|  |  |  | LGU81 | — | NMVZ10377 | S1 | HQ454898 |
| 48 | Snell’s Rd, 2 km S Wartook, Horsham Shire | VIC | LGU82 | — | NMVZ10378 | S1 | HQ454899 |
|  |  |  | LGU83 | — | NMVZ10379 | S1 | HQ454900 |
| 49 | 1.9 km N & 0.7 km NE Donovans | SA | LGU103 | SAMAR49371 | ABTC37418 | S1 | HQ454807 |
|  |  |  | LGU104 | SAMAR49372 | ABTC37419 | S1 | HQ454808 |
| 50 | 7.3 km E Carpenter Rocks | SA | LGU107 | SAMAR49559 | ABTC37504 | S1 | HQ454807 |
| 51 | Mary Seymour CP | SA | LGU111 | SAMAR33331 | ABTC14522 | S1 | HQ454816 |
|  |  |  | LGU112 | SAMAR33332 | ABTC14523 | S1 | HQ454817 |
| 52 | 17.6 km WSW Struan | SA | LGU105 | SAMAR49493 | ABTC37493 | S1 | HQ454809 |
|  |  |  | LGU106 | SAMAR49494 | ABTC37494 | S1 | HQ454810 |
| 53 | 10.2 km E &14.5 km NE Robe | SA | LGU108 | SAMAR49617 | ABTC37737 | S1 | HQ454812 |
|  |  |  | LGU109 | SAMAR49620 | ABTC37743 | S1 | HQ454813 |
| 54 | E of West Bay Rd, 7.5 km E of Cape Borda, Kangaroo I | SA | LGU4 | ANWC R5150 | ANWC R5150 | S2 | HQ454800 |
| 55 | 1.2 km NE D’Estree’s HS, Kangaroo Island | SA | LGU101 | SAMAR37211 | ABTC33698 | S2 | HQ454806 |
| 56 | 2.2 km ESE Deep Ck HS, Fleurieu Peninsula | SA | LGU115 | SAMAR45126 | ABTC58042 | S2 | HQ454820 |
|  |  |  | LGU116 | SAMAR45127 | ABTC58043 | S2 | HQ454821 |
| 57 | 21-22 km ESE Mt Compass | SA | LGU99 | SAMAR49209 | ABTC33244 | S2 | HQ454913 |
|  |  |  | LGU100 | SAMAR49210 | ABTC33245 | S2 | HQ454805 |
| 58 | 2.7 km NNW & 0.7 km SE Mt Lofty | SA | LGU126 | SAMAR54977 | ABTC74459 | S2 | HQ454832 |
|  |  |  | LGU127 | SAMAR54983 | ABTC74466 | S2 | HQ454833 |
| 59 | 5 km NE Lobethal | SA | LGU124 | SAMAR54895 | ABTC74377 | S2 | HQ454830 |
|  |  |  | LGU125 | SAMAR54911 | ABTC74380 | S2 | HQ454831 |
| 60 | Pewsey Vale, 6 km SE Tanunda | SA | LGU121 | SAMAR54085 | ABTC73291 | S2 | HQ454827 |
| 61 | Kaiser Stuhl CP | SA | LGU122 | SAMAR54812 | ABTC74207 | S2 | HQ454828 |
|  |  |  | LGU123 | SAMAR54843 | ABTC74226 | S2 | HQ454829 |
| 62 | Seven Hills, Flinders Ranges | SA | LGU91 | SAMAR30318 | ABTC14497 | S2 | HQ454907 |
| 63 | 7.2 km E Telowie | SA | LGU119 | SAMAR53273 | ABTC70487 | S2 | HQ454824 |
|  |  |  | LGU120 | SAMAR53274 | ABTC70498 | S2 | HQ454826 |
| 64 | 8.1 km SSW Wilmington | SA | LGU117 | SAMAR53192 | ABTC70358 | S2 | HQ454822 |
|  |  |  | LGU118 | SAMAR53193 | ABTC70359 | S2 | HQ454823 |
